# Is this the time to bid farewell to integrated pest management?

**DOI:** 10.3389/finsc.2026.1748643

**Published:** 2026-05-07

**Authors:** Ramasamy Srinivasan

**Affiliations:** Biological Sciences Research Program, World Vegetable Center, Tainan, Taiwan

**Keywords:** integrated pest management, consumer engagement, horizontal integration, institutional transformation, integrated plant health management, push-pull-policy (3P)

## Abstract

The concept of Integrated Pest Management (IPM) has been established for over five decades, primarily to reduce farmers’ dependence on harmful chemical pesticides. Numerous countries have allocated substantial financial resources towards research, piloting, and scaling IPM initiatives. However, the low adoption rates of IPM underscore a notable discrepancy between its theoretical constructs and practical implementation in agricultural settings. This perspective paper introduces a novel framework, ‘Integrated Plant Health Management’ (IPHEM), to bridge this gap. The IPHEM concept is developed within a ‘push-pull-policy’ (3P) architecture, positioning it as an essential component of sustainable crop production rather than merely a tool for pest and disease management. The ‘push’ aspect encompasses research activities related to discovery, piloting, and scaling IPHEM, as well as stakeholder engagement in its development and scaling. Conversely, the ‘pull’ aspect emphasizes the influence of consumer preferences and market demand for safe food as pivotal factors for the widespread adoption of IPHEM strategies in sustainable agriculture. The ‘policy’ dimension outlines the requisite enabling environment, regulatory frameworks, value chains for inputs and outputs, and infrastructure vital for effective deployment. Thus, this paper elucidates the interconnected, holistic nature of the 3P framework for operationalizing the IPHEM approach and advocates a shift away from traditional IPM models.

## 1 Introduction

After Rachel Carson alarmed the world with her book “Silent Spring,” published in 1962, awareness grew of the adverse impacts of chemical pesticides that modern agriculture had begun to adopt, affecting both the environment and human health. Although the foundations of integrated pest management (IPM) were starting to take root from the ‘supervised control’ of crop pests (1) through ‘Integrated Control’ (2), it did not become fully functional until the 1970s, when the Food and Agriculture Organization (FAO) and the International Organization for Biological Control (IOBC) started promoting it actively. For the FAO, Smith and Reynolds (3) defined IPM, a definition later adapted by the IOBC (4). Despite six decades of vigorous piloting and attempts to scale its use, adoption of IPM remains significantly low.

Several factors have been identified as barriers to the IPM adoption. They have been well researched (5) and reviewed (6). Interestingly, the IPM definition has undergone 67 variants over the past 40 years (7). Over two decades of my career at an international agricultural research center have allowed me to interact and collaborate with various stakeholders involved in IPM. This unique opportunity enabled me to grasp the hard realities behind low IPM adoption rates. IPM practitioners interpret and implement the concept quite differently across various regions of the globe, not surprisingly, given its multiple definitions. The knowledge gaps, skills, and perceptions about IPM among farmers are among the critical barriers (6). Largely, the approach to IPM is viewed as a “top-down” process, transferring knowledge from researchers to farmers through extensionists, rather than co-development involving end-users, which might help the uptake of knowledge-intensive technologies such as IPM. In 1989, when writing about the strengths and weaknesses of indigenous technical knowledge among farmers in Honduras, the cultural anthropologist Jeffery Bentley precisely described “What farmers don’t know can’t help them,” (8) which squarely fits the knowledge gap in IPM. Although the Farmer Field School (FFS) approach demonstrated success in narrowing this knowledge gap, it was not widely adopted for scaling IPM applications due to a lack of sustained resources.

The complexity of IPM is another obstacle to its adoption. Pesticides remain the predominant pest management tool to date (9) because of their straightforward action, which farmers easily understand and perceive as effective against perceived pest and disease losses. In addition, they are mostly applied through calendar-based spraying, because on-farm monitoring and threshold-based decision-making are almost absent. In the current context of production- and profitability-oriented agriculture, ecological and biological components described in IPM are perceived as additional, complex layers. Although host plant resistance is considered a major component of IPM, high-yielding cultivars with appreciable levels of resistance to pests and diseases are not widely available, particularly in developing countries. While the traditional IPM pyramid (10) amid is considered quite complex for farmers in developing countries, the recent IPM pyramid (11) and a new IPM paradigm (12) are even more complex. Both authors provide useful theoretical insights, but their introduction of high levels of conceptual density and their use of complex terms and fragmented bubbles can make it difficult for field practitioners and policymakers to follow. This complexity risks alienating those who need straightforward, actionable guidance. By complicating the internal mechanics of IPM, these models create ambiguity that surrounds the connection between biological interventions and the policy frameworks necessary to support them.

Furthermore, the IPM tool currently focuses on a narrow range of pests and diseases within a cropping system. Although the term pest encompasses insects, mites, pathogens, nematodes, vertebrates, and weeds, IPM is generally perceived as a management tool for insects and mites. Hence, phrases such as ‘integrated disease management’, ‘integrated weed management’, and ‘integrated nematode management’ are common, alongside IPM. Thus, horizontal integration of pest management components targeting various biotic constraints impacting crop productivity is uncommon in IPM practice to date, as evidenced by a call to shift from a pest-centric to a system-centric or holistic approach (11, 13). Compared to investment in discovery, product development, and marketing of chemical pesticides, IPM innovation, piloting, and scaling receive less attention and investment. For instance, the global market value of biopesticides is about US$9 billion, whereas the global chemical pesticide market is US$78 billion (14). The availability, accessibility, and affordability of IPM products, such as biopesticides and pheromones, are critical factors that hinder the adoption of IPM practices. Over the years, chemical pesticide groups have made significant advances and reached farmers (15), while biopesticides have not reached farmers widely, even a century after the discovery of *Bacillus thuringiensis*. This slow development can also be attributed to a lack of supportive policies and overly complicated regulatory processes (6, 16).

The focus on the ‘agroecosystem’ has been overlooked in current IPM practices. In response to calls for a stronger ecological emphasis in IPM, Deguine et al. (17) advocated for a shift towards agroecological crop protection (ACP). In their recent paper, Deguine et al. (6) further developed the concept of ACP. This approach is built on two main pillars: biodiversity and soil health. ACP aims to enhance the ecological health of agroecosystems by optimizing the interactions between living communities, both above and below ground. Although conceptually well-developed, many examples of ACP have focused primarily on vertical rather than horizontal integration. This is illustrated by studies on cotton insects (18), the cucurbit fruit fly (19), and mango pests (20). While ACP shows promise, it is still in the early stages of development and faces institutional and policy barriers similar to those encountered by IPM.

The acronym IPM has become deeply embedded in the minds of researchers, practitioners, farmers, the pest management industry, funders, and policymakers. Regardless of the specifics of different approaches, they will often be labeled as IPM, as Zalucki et al. (21) noted. Therefore, now is the perfect time to develop a conceptual framework that emphasizes the horizontal integration of pest management components, involving various stakeholders within a supportive policy environment. A conceptual shift is essential at this stage to align the expectations of end users and advocates, and to respond to a modern IPM fatigue by offering a streamlined, policy-anchored logic that matches the complexity of today’s global food systems.

## 2 Integrated plant health management

Integrated Plant Health Management (IPHEM) is introduced as a progressive paradigm that supersedes the conventional IPM framework. While IPHEM retains the foundational principle of ‘integration’, it redefines this concept specifically within the framework of plant health as a fundamental component of agronomic and crop management practices. This conceptual shift emphasizes plant health from the onset of field preparation through harvest, thereby establishing it as a preventive cultural component that integrates horizontally with traditional IPM control methods. Thus, IPHEM is defined as a holistic, plant-centric paradigm that redefines pest management as an intrinsic component of routine agronomic practices rather than a reactive crop protection strategy. It simplifies traditional methods into a streamlined, horizontally integrated framework of cultural, biological, and chemical components, operationalized through a push-pull-policy (3P) architecture.

Current IPM practitioners often view various methods primarily as pest management tools. In contrast, the IPHEM framework advocates a holistic approach to practice agronomic and crop management practices that can effectively mitigate the emergence of pests and diseases. This approach is designed to be less burdensome for farmers by integrating plant health strategies seamlessly into existing agronomic routines, making them feel less like an additional layer of crop protection and more like optimized farming practices. The operationalization of IPHEM is designed as a season-long activity that aligns with the crop’s specific phenological stages, ensuring proactive management of plant health from seeding to harvest. While annual crops require rapid, intensive synchronization of IPHEM components within a single growing season, perennial systems enable multi-year horizontal integration, in which long-term cultural practices and a stable biological equilibrium can be established over successive phenological cycles. The IPHEM framework streamlines this by simplifying the scope of conventional IPM, which typically encompasses host plant resistance and cultural, physical, mechanical, behavioral, biological, and chemical control methodologies. The inherent complexity and broad scope of the traditional IPM approach often inadvertently lead farmers to over-reliance on simple, immediate chemical interventions.

The IPHEM framework streamlines plant health methods into three core components: cultural, biological, and chemical. The biological component encompasses natural enemies, such as predators and parasitoids, as well as biopesticides, including microbial and botanical agents. In turn, the cultural component addresses all other agronomic practices, including field preparation, the selection of seed varieties, the use of healthy seeds or seedlings, water and nutrient management, weed control, and associated crops (mixed, inter-, and relay cropping). Notably, pheromones, other attractants, and insect growth regulators, which are fundamentally chemical in nature, are included in the chemical category because they function as specialized, purchased products that require specific application and management protocols, distinct from routine cultural practices. Overall, IPHEM is articulated as a concept with a straightforward structural framework.

**Figure 1:**
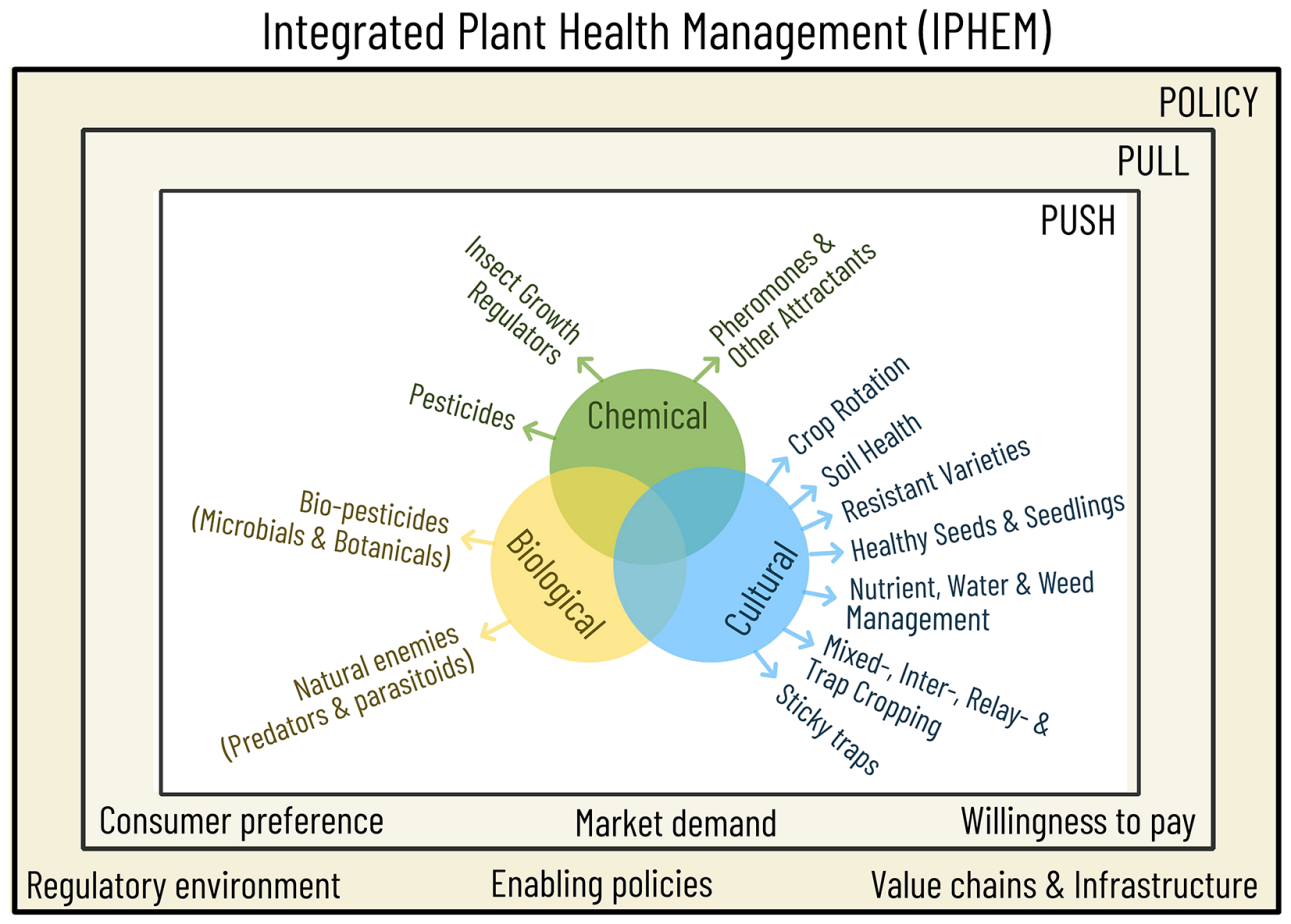
The Integrated Plant Health Management (IPHEM) Framework. The IPHEM framework represents a strategic shift from a narrow, pest-centric, vertically integrated approach to a more comprehensive, plant-centric, horizontally integrated approach. This framework aims to bridge the “Death Valley” gap that exists between discovery research and farmer adoption. It seeks to integrate the entire value chain, supported by market demand for safe food and favorable policy environments.

At the heart of this new paradigm lies the “Push, Pull, and Policy” (3P) framework, a cornerstone of the IPHEM concept, as illustrated in **Figure 1**. This framework aims to integrate plant health as an intrinsic component of crop production throughout the entire cycle, from planning to harvest focusing on biotic constraints as a whole, contrary to the prevailing perception of IPM, which farmers and extensionists predominantly view as a reactive crop protection strategy. The 3P framework underscores the equal significance of three aspects: the promotion and facilitation of plant health innovations (push), the influence of consumer demand on their uptake (pull), and the establishment of a supportive policy environment (policy). The foundational structure of IPHEM, encompassing cultural, biological, and chemical components, aligns with the ‘push’ dimension, as practitioners will present these methods as technological advancements aimed at preventing pests and diseases or managing them, while exceeding threshold levels through horizontal integration.

The ‘pull’ aspect is operationalized through market mechanisms that incentivize the adoption of IPHEM practices. In contrast, traditional IPM approaches often lack economic inducements, rendering the high production costs of safe produce unsustainable. The prevailing market dynamics do not differentiate between products cultivated under IPM and those from non-IPM systems. Only a minor segment of niche markets and a small proportion of health-conscious consumers are prepared to pay a premium for produce from IPM farms (22–24). The IPHEM framework addresses this disparity by operationalizing the ‘pull’ aspect through tangible economic incentives for produce originating from IPHEM-compliant farms. This objective may be achieved through enhanced consumer awareness and the establishment of connections with niche markets as integral components of IPHEM initiatives. The increasing demand for products derived from IPHEM practices and the existence of niche markets will incentivize broader adoption among farmers.

Moreover, the third crucial element of the 3P framework is policy. Policymaking spans a wide range and requires coordination across sectors such as agriculture, health, trade, and finance. For effective implementation of both the push and pull strategies, a conducive policy environment is imperative. For instance, a nation must cultivate an enabling policy landscape that fosters the discovery, development, and commercialization of diverse technological innovations within the push aspect. While it is acknowledged that investments in alternative methods may not rival those of the pesticide industry, it is critical to avoid lengthy delays in the discovery/exploration and development of innovative IPHEM tools, such as complementary microbial agents to *Bacillus thuringiensis*, RNAi-based biopesticides, precision bacteriophages for bacterial disease suppression, and advanced semiochemical systems for area-wide pest regulation. To accelerate this transition, policy interventions must include regulatory sandboxes and fast-track registration pathways for low-risk biologicals to lower entry barriers for SMEs, as well as incentives for decentralized, local-level production hubs to overcome infrastructure and cold-chain limitations. Additionally, policies should mandate technology transfer from public research to local startups and prioritize ‘bio-first’ strategies in national extension services to ensure these innovations become the primary recommendation for farmers. Finally, the pull aspect requires supportive policy frameworks at all levels of government, from local municipalities to national administrations, to effectively safeguard citizens’ health. Specifically, policy interventions are imperative to provide economic incentives and niche-market opportunities for IPHEM adopters, and to increase consumer awareness to create demand for safe produce from IPHEM-compliant farmers.

## 3 Discussion

Twenty-five years ago, I started my career as a passionate advocate for IPM. Throughout the years, I’ve noticed a common misconception that IPM can fully replace chemical pesticides. I have aimed to promote IPM alongside good agricultural practices, organic farming, agroecological methods, and regenerative agriculture to achieve not only chemical-free farming but also safe, sustainable practices. This includes the optimal use of agrochemicals when necessary. Despite similar efforts by thousands of IPM practitioners worldwide, the adoption rate of IPM remains historically low for various reasons (5).

This paper does not aim to discuss the constraints and barriers to IPM adoption. Instead, I propose a practical approach to reduce reliance on chemical pesticides in agriculture and alleviate the fatigue surrounding IPM among stakeholders, including practitioners, farmers, industries involved in IPM products, policymakers, and funders. The proposed concept, IPHEM, is not merely a new label for traditional IPM; it retains the essential components of IPM while presenting them in a simpler, more understandable manner for key stakeholders such as farmers, extension agents, and policymakers.

IPHEM consists of three main layers. The innermost layer includes the “push” components, which maintain most IPM elements but are organized more clearly. These components encompass crop production and management practices aimed at preventing pest and disease outbreaks. They also highlight the need for additional inputs that are currently unavailable, inaccessible, or unaffordable for farmers. For example, alternative solutions such as pheromones and biopesticides are not widely available and accessible; the choices are limited and do not adequately address the primary pests and diseases affecting smallholder farmers (25).

For instance, despite the discovery of *Bacillus thuringiensis* over a century ago, there has yet to be a complementary bacterial biopesticide for managing lepidopteran pests. Although some entomopathogenic fungi have been used in IPM frameworks, their effectiveness varies depending on the specific pests and prevailing climatic conditions (26). Therefore, increased investment in research is crucial for diversifying biopesticide options and developing innovative formulations with longer shelf life, greater efficacy, compatibility, and synergy with other pest and disease management strategies. We need to strengthen agro-input value chains for alternative solutions, as we have done with chemical pesticides, to ensure farmers can easily access these resources. It is also essential to encourage multiple players to enter the production, distribution, and marketing of alternative products included in IPHEM packages, helping to keep prices affordable for smallholder farmers.

All of these changes can occur only with a supportive policy environment at the local, national, and regional levels, which has been a critical bottleneck for IPM (16). Hence, the overarching layer of the IPHEM concept represents the policy framework that encompasses both the push and pull components. Without an enabling policy environment and a solid regulatory framework, innovative technologies and products for pest and disease management risk remaining confined to academic publications, failing to reach farmers for decades or even a century.

A demand-driven framework is imperative for the widespread adoption of any innovation, and IPM is no exception. Despite over fifty years since its introduction and advocacy, the adoption of IPM has not attained the anticipated levels. In contrast to organic agriculture (27) and Good Agricultural Practices (GAP) (28, 29), there is currently no universal ‘IPM label’ within the marketplace. This lack of standardized labeling has prevented the market from distinguishing between products from conventional farming practices and those from IPM-adopted farms. Consequently, there are insufficient economic incentives for growers to adopt IPM strategies. Furthermore, producers who adopt IPM often face economic challenges because IPM products tend to cost more than conventional chemical pesticides.

To facilitate increased adoption of IPM, it is essential to establish economic incentives that reward the production of safe products from IPM farms. The proposed IPHEM concept incorporates this economic element within the ‘pull’ layer of its framework. Organized crop production and marketing initiatives, such as farmers’ associations, cooperatives, agri-business clusters, and vegetable business networks, are either currently operational or in the early stages of development globally, particularly in the developing world. The implementation of the IPHEM concept can thus be effectively institutionalized through these organized production systems.

Additionally, enhancing consumer awareness regarding the benefits of safe produce will further stimulate demand, thereby supporting individual farmers who adopt IPHEM practices in their production processes. However, it is crucial that the policy layer actively supports the pull layer, as an enabling policy environment is necessary for the successful implementation of certification and labeling processes and for establishing value chains and niche markets for IPHEM products. This underscores the importance of integrating the policy layer as a fundamental component of the IPHEM concept.

## 4 Conclusions

In conclusion, the persistent challenges surrounding the IPM adoption highlight the need for a paradigm shift towards Integrated Plant Health Management (IPHEM). This innovative approach addresses the gaps between theoretical frameworks and practical implementation by embracing a holistic ‘push-pull-policy’ (3P) architecture. By integrating research, stakeholder engagement, and market while labor dynamics, IPHEM offers a sustainable pathway for crop production that prioritizes consumer preferences for safe food while fostering collaboration and knowledge sharing among farmers. It is crucial to move beyond traditional IPM models and adopt strategies that account for the complexities of agricultural ecosystems and the real-world challenges farmers face. By doing so, we can enhance the efficacy and uptake of sustainable plant health approaches that ensure both environmental health and food security.

## Data Availability

The original contributions presented in the study are included in the article/supplementary material, further inquiries can be directed to the corresponding author/s.
